# What Will Be the New Standard of Morals?

**Published:** 1913-10

**Authors:** Thos. S. Blair

**Affiliations:** Harrisburg, Pa.


### Treatment of Cranial Injuries

By FRANK W. McGUIRE, Attending Surgeon to Emergency Hospital.
Lecturer in Surgery at University of Buffalo.

INJURIES so rich in complications as those of the skull naturally present difficult problems in their manner of treatment. There is no injury which necessitates such a careful balancing of all the details of each separate case. Injuries to the blood supply, to the brain tissue, and the later inflammatory or degenerative processes that occur, must be severally and collectively considered in forming conclusions as to the proper treatment.

It is unfortunate that these injuries are so universally classed as fractures, for this leads to undue attention upon the lesion of the bone, to the exclusion of that of the brain. It should not be forgotten, however, that violence which causes fatal injury of the brain, with fracture of the skull, may, under slightly changed conditions, cause the brain injury without the fracture. In a large proportion of cases the fracture is merely an incident, without any direct relation to the unhappy result, or only that of having made the causative lesion possible.

I have endeavored to group injuries of the brain under two headings; first, those of the base; secondly, those of the vault. In injuries of the base, we usually have a fracture extending to and occupying the base of the skull and hemorrhages covering a large area of brain. In injuries to the vault, the lesions are usually local, limited to the bone and overlying soft parts. In these injuries the fracture of the bone is the essential lesion and the treatment is almost entirely directed to it. This class of fracture is usually compound, with a circumscribed depression of the vault, possibly with a rent in the dura. Besides the fractures of the base and vault we often see both combined, the one extending to the other.

As to the treatment of the second group in which the lesion is entirely local, it is usually limited to the bone and overlying soft parts. These injuries are usually produced by a blow from a relatively small body or one having an edge or corner, the fracture is compound, and diagnosis is made by inspection and palpation. When the bone is distinctly broken and depressed it should be raised. If the fracture includes only the outer table, the operation need not be carried further, but, if the entire thickness of bone is broken the deeper as well as the superficial should be raised. If the dura is torn it should be closed, provided there is not profuse bleeding from pia. When, however, there is bleeding from the pia, it should be controlled either by a circular suture of fine catgut or packed with gauze. Drainage, however, should not be used, except in uncontrollable hemorrhages, because it predisposes infection. When the bone is comminuted, care should be used not to separate it from its periosteum, so as to preserve the bone and thereby save the contour of the skull.

This class of injuries usually make good recoveries if sepsis can be avoided. The reason for the recoveries in this group of cases is accounted for by the fact that they do not suffer from cerebral compression as the injury has performed a decompression in the nature of a compound fracture.

In small perforations caused by nail or pistol shot, the opening should be enlarged for better cleansing and removal of small fragments of bone. Sometimes troublesome bleeding occurs; this should be controlled. Bullets should not be removed unless easy of access, as a stationary bullet causes little damage; the real damage being caused by the bullet in its flight.

The symptoms of the first group, i.e., those in which the injury extends to, and occupies the base, with a lesion of the brain, will vary with the extent of the injury. The chief symptoms are: Unconsciousness, either immediate or later; irregularity of pupils, slow pulse, increased blood pressure, rise of temperature, restlessness and sometimes irritation of various groups of muscles. Symptoms referring to respiration and circulation depend upon the portion of the brain involved. Blood is almost always present in lumbar puncture if the injury is subdural with hemorrhage, but its absence does not preclude extra-dural hemorrhage when the dura is not injured. Hemorrhages from the ear, nose and mouth are highly indicative of fracture of the base, and positively so if free cerebro spinal fluid is present. This hemorrhage is, in a number of cases, life-saving, as it tends to lessen endocranial pressure.

Ecchymosis beneath the conjunctiva, not due to local bruising, is usually associated with fracture of the orbital plate and spenoid.

In the event of a watery discharge from the ear, the flow being abundant and prolonged, containing little albumen and large quantities of sodium chloride, the fluid will be cerebrospinal. If it be highly albuminous without sodium chloride the fracture is through the internal auditory canal, the discharge coming from the large arachnoid lymph spaces in the ear. Deafness of the affected side is due to injury of the middle, internal ear or the nerve in its passage through the bone.

Paralysis of the cranial nerves is due to direct injury of the nerve or to intracranial blood pressure. Paralysis of the limbs is due to hemorrhage causing pressure on the motor areas.

In discussing the treatment of fractures of the base, let me quote a recent text-book:

"The treatment of these fractures is medical and expectant; absolute rest, light diet, laxatives, cold to head if indicated by headache, restlessness, etc."

There is no question but that the treatment of certain cranial injuries is palliative, and the chances for ultimate recovery are increased by no operative methods. Such cases, however, require careful selection. We can say at least that such cases have slight endocranial pressure and loss of consciousness is usually of short duration. There is, however, another class of cases that die of endocranial pressure, and if these could be operated on early enough to prevent serious injuries to the brain, undoubtedly some of them would recover. When severe pressure exists, it will appear quite plain that palliation has little to recommend it. The surgeons have taught the internist most of what he knows about the appendix, gall-bladder, stomach, duodenum and pancreas. The same story will be true of the brain. The surgeons, by repeated operations, will learn more about the physiology and pathology of the brain, and will learn how and when to operate. Indeed there is an increasing number of surgeons who believe in early relief of compression, and the operation for cranial injuries are increasing. A flap of bone should be elevated on one or both sides of the head. An inverted flap may be used, as it gives a larger exposure in which to cope with the blood clots or hemorrhage on the under surface of the brain. Hemorrhage of the base should be dealt with directly when possible. When impossible one of the decompression operations should be performed.

If decompression is necessary the operation suggested by Hudson or E. R. McGuire will give the best result. In doing brain surgery it is necessary to have a full set of Hudson's instruments.

1—Keen's Surgery.

2—Modern Surgery—Park.

3—Treatment of fractures—Scudder.

4—Fractures and dislocations—Stimson.

---

A Case of Pulsus Alternans In the Course of a Chronic Nephritis. G. Régnier, (*Arch. des Mal du Coeur, des Vaisseaux, et du Sang,* 1913, VI, 97). The author gives the clinical history and polygraphic curves of the pulse in a case of chronic interstitial nephritis with pulsus alternans. There was secondary cardiac hypertrophy. The pulsus alternans appeared during an attack of decompensation and was followed by the death of the patient.

